# Prognostic role of expression of N-cadherin in patients with upper tract urothelial carcinoma: a multi-institutional study

**DOI:** 10.1007/s00345-016-1968-2

**Published:** 2016-11-09

**Authors:** Mohammad Abufaraj, Marco Moschini, Francesco Soria, Kilian Gust, Mehmet Özsoy, Romain Mathieu, Morgan Rouprêt, Vitaly Margulis, Jose A. Karam, Christopher G. Wood, Alberto Briganti, Karim Bensalah, Andrea Haitel, Shahrokh F. Shariat

**Affiliations:** 1 Department of Urology, Vienna General Hospital, Medical University of Vienna, Vienna, Austria; 2 Department of Urology, Urological Research Institute, San Raffaele Scientific Institute, Vita-Salute University, Milan, Italy; 3 Division of Urology, Department of Surgical Sciences, San Giovanni Battista Hospital, University of Studies of Torino, Turin, Italy; 4 Department of Urology, University of Rennes, Rennes, France; 5 Department of Urology, Pitié-Salpétrière, Assistance-Publique Hôpitaux de Paris, Faculté de Médecine Pierre et Marie Curie, University Paris VI, Paris, France; 6 Department of Urology, University of Texas Southwestern Medical Center, Dallas, TX USA; 7 Department of Urology, The University of Texas M.D. Anderson Cancer Center, Houston, TX USA; 8 Department of Pathology, Medical University of Vienna, Vienna, Austria; 9 Karl Landsteiner Institute of Urology and Andrology, Vienna, Austria; 10 Department of Urology, Weill Cornell Medical College, New York, NY USA

**Keywords:** N-Cadherin, Urothelial carcinoma, Upper tract urothelial carcinoma, UTUC prognosis, Survival, Prediction

## Abstract

**Purpose:**

To assess the role of N-cadherin as prognostic biomarker in patients with upper tract urothelial carcinoma (UTUC) in a large multi-institutional cohort of patients.

**Patients and methods:**

Immunohistochemistry was used to evaluate the status of N-cadherin expression in 678 patients with unilateral sporadic UTUC treated with radical nephroureterectomy. N-cadherin was considered positive if any immunoreactivity with membranous staining was detected. The Kaplan–Meier method was used to estimate recurrence-free survival, overall survival and cancer-specific survival. Disease recurrence, overall mortality and cancer-specific mortality probabilities were tested in Cox regression models.

**Results:**

Expression of N-cadherin was observed in 292 (43.1%) of patients, and it was associated with advanced tumour stage (*p* < 0.04), lymph node metastases (*p* = 0.04) and sessile architecture (*p* < 0.02). Within a median follow-up of 37.5 months (IQR 20–66), 171 patients (25.2%) experienced disease recurrence and 150 (22.1%) died from UTUC. In univariable analyses, N-cadherin expression was significantly associated with higher probability of recurrence (*p* = 0.01), but not overall (*p* = 0.9) or cancer-specific mortality (*p* = 0.06). When adjusted for the effects of all available confounders, N-cadherin was not associated with any of the survival outcomes.

**Conclusion:**

N-cadherin is expressed in approximately 2/5 of UTUs. It is associated with adverse pathologic factors but not with survival outcomes. Its clinical value remains limited.

**Electronic supplementary material:**

The online version of this article (doi:10.1007/s00345-016-1968-2) contains supplementary material, which is available to authorized users.

## Introduction

Upper tract urothelial carcinoma (UTUC) is a relatively rare entity that represents 5–10% of Urothelial carcinoma (UC) with an estimated incidence of 1–2 cases per 100,000 inhabitants in western countries [1, 2]. It is still considered an aggressive disease with a high incidence of disease progression and mortality [3, 4]. Radical nephroureterectomy (RNU) with bladder cuff excision remains the treatment of choice for non-metastatic UTUC [2]. Since perioperative chemotherapy has an essential role in high-risk patient and conservative treatment allows the preservation of renal functional unit in low-risk patient [2, 5], accurate risk stratification is essential for patient counselling, treatment planning and follow-up scheduling [6]. Current prognostic and predictive tools based on standard clinicopathological factors remain unfortunately insufficient to yield enough accuracy for clinical decision-making [4, 7]. Molecular markers associated with clinically significant outcome may help improve our current prognostication.

Cadherins are transmembrane glycoproteins that play a central role in cell–cell adhesion in epithelial tissue [8]. N-cadherin is not expressed by normal urothelium but it has been demonstrated that N-cadherin expression is associated with more invasive phenotype in urothelial carcinoma and other malignancies [8–10]. Several investigators evaluated the prognostic value of different tissue-based markers on UC, but only few investigated cadherins’ role in UTUC [11–13].

We hypothesized that expression of N-cadherin in RNU specimens is associated with more invasive and aggressive phenotype and affects oncological outcomes. To assess this, we tested the association of N-cadherin with pathologic characteristics and prognosis in a large multi-institutional cohort of patients treated by RNU for UTUC.

## Materials and methods

### Patient selection

This is a multi-institutional retrospective study involving eight centres from Europe and North America from the international UTUC collaboration [14]. All participating sites obtained institutional review board approval for the study and provided institutional data-sharing agreements before the initiation of the study. The initial study cohort comprised 753 patients who underwent RNU for UTUC between March 1990 and May 2008. Patients who underwent neo-adjuvant chemotherapy/radiotherapy (*n* = 19) and patients with follow-up duration of <3 months (*n* = 56) were excluded from the study. A total of 678 patients were included in the final analysis.

### Data collection, pathological evaluation and immunohistochemistry

Clinical, pathologic and follow-up data were collected from patients’ medical records. Original slides were collected and reviewed by two experienced genitourinary pathologists blinded to clinical outcome to ensure the validity of pathological data extraction [15]. Pathological stage was determined according to the 2002 tumour, node and metastasis (TNM) staging system, and tumour grade was evaluated in accordance with 1973 WHO grading system for patients before 2005 and with both 1973 and 2004 WHO grading system for specimens collected from 2005 to 2008 [16]. Tumour architecture was defined as papillary or sessile [17]. Lymphovascular invasion (LVI) [18], multifocal tumour [7], carcinoma in situ and tumour necrosis [19] were confirmed in every patient. Tumour necrosis in more than 10% of tumour area was considered positive for clinicopathological association [19].

N-cadherin immunohistochemical staining was performed on formalin-fixed tissue microarray slides in a single laboratory with 3 cores per patients evaluated. Four-µm tissue microarray sections were deparaffinized by xylene, rehydrated in graded alcohols, treated with 1% hydrogen peroxide and submitted to heat-induced epitope retrieval (Dako Epitope Retrieval Solution, 40 min, 98 °C). Subsequently, antigen retrieval was performed and the primary anti-N-CD monoclonal mouse antibody (Transduction Labs, dilution 1:50 in blocking solution) was incubated for 1 h. Secondary antibody (Vector Labs) was applied at a dilution of 1:400. Reactivity was visualized with an avidin–biotin complex immunoperoxidase system using diamino-benzidine as the chromogen and methyl green and alcian blue as the counterstain. Positive controls included bladder and prostate tissue known to possess N-cadherin expression (external control). Negative controls were serial sections processed without incubation in primary antibody. E-cadherin immunohistochemical staining has been previously described [20].

### Management and follow-up

Before surgery, all patients underwent full clinical evaluation including history, physical examination, blood tests and appropriate imaging study/studies (computed tomography, magnetic resonance imaging and chest x-ray). All patients underwent RNU [2], but approach was not standardized. A regional lymphadenectomy was not routinely performed. Postoperative follow-up was generally performed every 3 months in the first year after surgery, every 6 months in the second year and annually thereafter. Relapse was defined by local recurrence or distant metastasis. Cause of death was determined by chart review or death certificate [21].

### Statistical analyses

Chi-square test was used to assess N-cadherin expression with categorical variables. Differences in continuous variables were analysed using Mann–Whitney *U* test. The Kaplan–Meier method was used to estimate recurrence-free survival (RFS), cancer-specific survival (CSS) and overall survival (OS); log-rank tests were applied for pairwise comparison of survival. Univariable and multivariable Cox regression models addressed associations of disease recurrence, cancer-specific mortality and overall mortality with potential prognostic factors. All *p* values were two-sided, and statistical significance was defined as *p* < 0.05. Statistical analyses were performed using Stata 11.0 statistical software (StataCorp., College Station, TX, USA).

## Results

### Descriptive characteristics and association with N-cadherin status

**Fig. 1:**
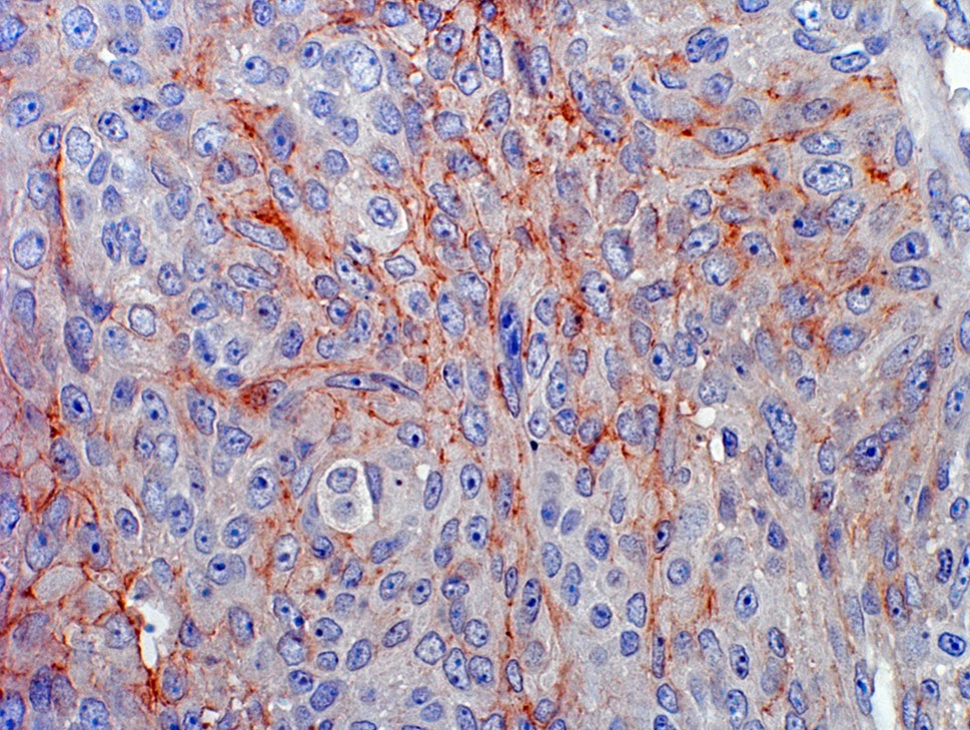
Typical outcome of immunohistochemical staining of primary urothelial carcinoma of the upper urinary tract with N-cadherin antibody

Overall, 57% of patients were men and the median age at time of surgery was 69 years (IQR 62-76). Expression of N-cadherin was observed in 292 patients (43.1%) (Fig. 1). Most patients underwent open RNU (78.5%), lymphadenectomy was performed in 22.9% of patients, and adjuvant chemotherapy was given in 10% of patients. Tumours were solitary in 78.6% of cases and located in the pelvicalyceal system in 70.5% of cases. Expression of N-cadherin was associated with pathological features such as advanced tumour stage (*p* = 0.04), lymph node metastases (*p* = 0.04) and sessile architecture (*p* < 0.02) (Table 1).

**Table 1:** Descriptive characteristics for the cohort of 678 patients with upper tract urothelial carcinoma treated with radical nephroureterectomy

Variables	Total	N-cadherin status		*p* value
		Negative	Positive	
Number of patients *n* (%)	678 (100)	386 (56.9%)	292 (43.1%)	
Median age (IQR), years	69 (63–76)	70 (62–76)	69 (63–76)	0.1
Gender, *n* (%)				0.7
Female	298 (44)	167 (43)	131 (45)	
Male	380 (56)	219 (57)	161 (55)	
Previous bladder cancer, *n* (%)	247 (36)	135 (35)	112 (38)	0.4
Side, *n* (%)				0.8
Right	307 (45.3)	176 (45.6)	131 (44.9)	
Left	371 (54.7)	210 (54.4)	161 (55.1)	
Type of surgery, *n* (%)				0.4
Open	532 (78.5)	307 (79.5)	225 (77)	
Laparoscopy	146 (21.5)	79 (20.5)	67 (23)	
Lymphadenectomy, *n* (%)	155 (22.9)	82 (21.2)	73 (25)	0.2
Tumour location, *n* (%)				0.001
Pelvicalyceal	478 (70.5)	291 (75.4)	187 (64)	
Ureter	200 (29.5)	95 (24.6)	105 (36)	
Tumour architecture, *n* (%)				0.02
Papillary	558 (82.3)	329 (85.2)	229 (78.4)	
Sessile	120 (17.7)	57 (14.8)	63 (21.6)	
Multifocal tumour, *n* (%)	145 (21.4)	82 (21.2)	63 (21.6)	0.9
Pathological tumour stage, *n* (%)				0.04
pTa, pTis	121 (17.8)	78 (20.2)	43 (14.7)	
pT1	208 (30.7)	127 (32.9)	81 (27.7)	
pT2	123 (18.1)	61 (15.8)	62 (21.2)	
pT3	193 (28.5)	106 (27.5)	87 (29.8)	
pT4	33 (4.9)	14 (3.6)	19 (6.5)	
Concomitant CIS, *n* (%)	128 (18.9)	63 (16.3)	65 (22.3)	0.05
Lymph node metastases, *n* (%)	49 (7.2)	21 (5.4)	28 (9.6)	0.04
Grade, *n* (%)				0.2
Low	174 (25.7)	107 (27.7)	67 (23)	
High	504 (72.3)	279 (72.3)	225 (77)	
Lympho-vascular invasion, *n* (%)	135 (19.9)	69 (17.9)	66 (22.6)	0.1
Necrosis, *n* (%)	81 (12)	42 (10.9)	39 (13.4)	0.3
Adjuvant chemotherapy, *n* (%)	68 (10)	34 (8.8)	34 (11.6)	0.2
E-cadherin, *n* (%)	353 (52.1)	194 (50.3)	159 (54.5)	0.3

*CIS* carcinoma of situ

Concordance between E-cadherin and N-cadherin expression status was 45% (supplementary Table 1).

### Survival analyses

**Fig. 2:**
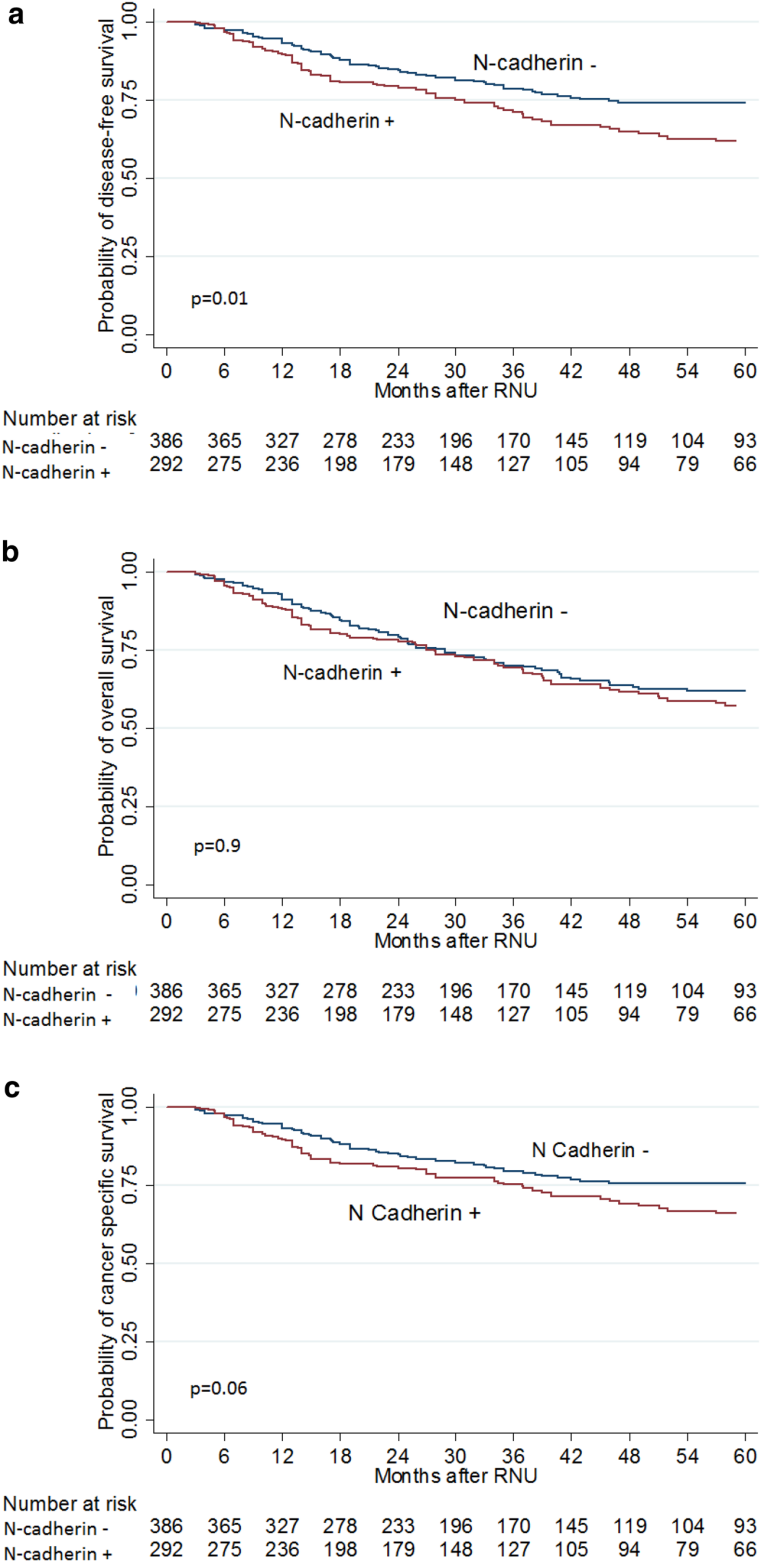
Kaplan–Meier estimates for **a** disease-free survival, **b** overall survival and **c** cancer-specific survival according to N-cadherin status in 678 patients treated with radical nephroureterectomy for upper tract urothelial carcinoma

The median follow-up time was 37.5 months (IQR 20–66). During this period, 171 patients (25.2%) experienced disease recurrence with median time to recurrence of 12 months (IQR 5-22); 234 deaths (34.5%) were recorded, of which 150 (22.1%) were caused by UTUC. Kaplan–Meier analysis revealed that patients expressing N-cadherin had a lower probability RFS, (log-rank test *p* = 0.01; Fig. 2a) compared to those without N-cadherin expression; this was not true for either OS (*p* = 0.9; Fig. 2b) or CSS (*p* = 0.06; Fig. 2c).

At univariable cox regression analyses, expression of N-cadherin was associated with higher probability of recurrence (HR 1.44, 95% CI 1.07–1.95, *p* = 0.016), but not overall mortality (*p* = 0.9) or cancer-specific mortality (*p* = 0.06). Table 2 summarizes the multivariable cox regression analyses predicting these outcomes. On multivariable analyses that adjusted for the effects of standard clinicopathological variables, N-cadherin expression was not associated anymore with probabilities of recurrence (*p* = 0.6), overall mortality (*p* = 0.2) or cancer-specific mortality (*p* = 0.9). Removal of E-cadherin from the multivariable analyses did not change the lack of statistical significance of N-cadherin with survival outcomes (data not shown).

**Table 2:** Multivariable Cox regression analyses predicting disease recurrence, overall and cancer-specific mortality of 678 patients treated with radical nephroureterectomy for upper tract urothelial carcinoma

Variable	Disease recurrence			Overall mortality			Cancer-specific mortality		
	HR	95% CI	*p*	HR	95% CI	*p*	HR	95% CI	*p*
Age (continuous)	1.02	1.01–1.04	0.004	1.04	1.03–1.06	<0.001	1.02	1.01–1.04	0.004
Female gender	0.71	0.52–0.97	0.036	0.92	0.71–1.20	0.6	0.77	0.55–1.07	0.1
Sessile architecture	1.33	0.90–1.97	0.1	1.28	0.91–1.81	0.2	1.41	0.93–2.14	0.1
pT stage									
(ref.: pTa, pTis)									
pT1	1.67	0.70–3.97	0.2	1.02	0.58–1.80	0.9	1.44	0.56–3.72	0.4
pT2	3.49	1.48–8.22	0.004	1.54	0.86–2.74	0.1	3.42	1.36–8.61	0.009
pT3	6.8	3.00–15.53	<0.001	2.60	1.50–4.51	0.001	5.96	2.45–14.49	<0.001
pT4	27.3	11.7–81.0	<0.001	9.17	4.47–18.79	<0.001	22.7	7.9–61.71	<0.001
pN + stage	2.21	1.43–3.39	<0.001	1.94	1.27–2.90	0.002	2.37	1.52–3.69	<0.001
Concomitant CIS	1.35	0.92–1.99	0.1	1.13	0.81–1.63	0.4	1.10	0.72–1.68	0.7
High grade	1.33	0.79–2.24	0.3	1.63	1.04–2.48	0.03	1.52	0.85–2.72	0.1
Lymphovascular invasion	1.17	0.81–1.69	0.4	1.23	0.90–1.74	0.2	1.32	0.89–1.94	0.2
Necrosis	0.50	0.30–0.81	0.005	0.78	0.53–1.19	0.3	0.51	0.31–0.85	0.01
Multifocal	1.61	1.12–2.31	0.009	1.59	1.17–2.15	0.002	1.76	1.20–2.56	0.003
E-cadherin	1.01	0.73–1.41	0.9	0.99	0.74–1.31	0.9	0.95	0.67–1.36	0.8
N-cadherin	1.09	0.79–1.51	0.6	0.83	0.64–1.12	0.2	1.01	0.72–1.43	0.9

*CI* confidence interval, *HR* hazard ratio, CIS carcinoma in situ

## Discussion

In all malignancies, efforts to identify factors associated with disease recurrence and survival outcomes are of utmost importance to set up treatment plans and follow-up schedules. In UTUC, precise staging cannot be made until RNU is performed; at that point, a proportion of patient have already missed the chance to get neo-adjuvant chemotherapy and/or regional lymphadenectomy which they may have benefited from. The challenge lies in the appropriate risk stratification of patients based on standard clinicopathological features, together with predictive and prognostic biomarkers [4, 22].

Several investigators studied the prognostic value of various tissue-based markers in UTUC such as p53, Ki67, EGFR, Snail, Bcl-2, Survivin, β-Catenin and E-cadherin [4]. To test the value of one biomarker for this purpose, we performed a retrospective multicenter study in which we evaluated the association of N-cadherin expression status with clinicopathologic features and prognostic outcomes in 678 patients treated with RNU for UTUC. We found that more than two-fifths of patients in this cohort presented with abnormal expression of N-cadherin and that expression was associated with adverse pathological features but not survival outcomes.

To the best of our knowledge, only one single-centre study evaluated the role of N-cadherin in UTUC [13]. In contrast to this study that included only 59 patients, we assessed the role of N-cadherin in a large international cohort comprising 678 patients. Muramaki et al. [13], studied the role of N-cadherin as predictor of intra-vesical and extra-vesical recurrence but they did not evaluate other oncological outcomes such as CSS and OS. We found that the expression of N-cadherin is associated with features of biologically and clinically aggressive UTUC such as advanced T stage and node metastasis. All these factors have been previously shown to be independently associated with poor survival [2, 4, 6, 23]. While association with pathologic factors is important, only association with survival outcomes will change management of UTUC patients and we failed to demonstrate an association of N-cadherin expression with oncological survival outcomes on multivariable analysis, limiting its prognostic value in clinical practice. In comparison with reports evaluating the association between N-cadherin and urothelial carcinoma of bladder, the percentage of expression of N-cadherin in UTUC in this study was within the range detected in bladder cancer reports [24–26] and lower than the previously reported in UTUC (43 vs 68%) [13]. Such discrepancies may be due to differences in staining and case mix of the population at hand. Moreover, the expression of N-cadherin in bladder cancer reports was higher in muscle invasive bladder cancer compared to non-muscle invasive bladder cancer. Normal urothelium did not show N-cadherin expression [8].

Since UC is a heterogeneous disease with complex underlying molecular mechanism, multiple biomarkers should be integrated into prognostic schemes to accurately guide our decision-making process [27–29]. In epithelial malignancies, epithelial cells undergo series of changes in morphology, adhesion and migratory capacity turning them into cells with mesenchymal characteristics, this process is called epithelial mesenchymal transitional (EMT) [30]. Many investigators studied more than one biomarker simultaneously [29], demonstrating the process of downregulation of epithelial markers and upregulation of mesenchymal markers, named as “cadherin switch” [8, 14]. Here comes the importance of studying other potential candidates implicated in carcinogenesis and invasive properties of urothelial carcinoma such as E and/or P-cadherin. For example, Muramaki et al. [13] addressed the effect of both N and E-cadherin expression on intra- and extra-vesical recurrence after RNU and they found that decrease in expression of E-cadherin and increase expression of N-cadherin is an independent prognostic factor for disease recurrence, a finding that goes in line with the cadherin switch concept. Also, several transcriptional factors play a role in suppression of epithelial markers and promotion of mesenchymal markers like snail, vimentin, slug and twist. These factors may also be used for prognostication or evaluated as a potential candidate for targeted therapy.

Since EMT process involves multiple markers and transcriptional factors in dynamic fashion, we analysed the data by making subgroup according to cadherin status. Patients with negative expression of E-cadherin were evaluated for possible association between N-cadherin and survival outcomes; no such association could be made. Similarly, in patients with positive E-cadherin expression association between N-cadherin and survival outcomes were assessed but also no such association could be made (supplementary Tables 1 and 2). In addition, removal of E-cadherin on multivariable cox regression analyses did not significantly change the statistical association between N-cadherin and survival outcomes.

Although we have found limited role of N-cadherin in prognostication and decision-making, the promise lies in other markers that could serve our main goal in improving the pre-operative risk stratification of patients with UTUC. Tissue-based, blood-based, urinary and genetic markers are collectively the fields for future research [4]. For tissue-based makers, exploration of biomarkers related to cellular processes such as cell adhesions, angiogenesis and apoptosis is an essential need not only for prognostication but also for identifying possible therapeutic targets.

This study is not without limitations; the most obvious is the retrospective and multicenter nature of data collection which may lead to inconsistencies in surgical technique, staging and laboratory evaluation that may cofound outcomes. Additionally, to incorporate molecular markers into prognostic model, a prospective study is required. The second limitation is the reliability of immunohistochemical technique which is semiquantative and depends on a range of variables such as fixation techniques, preservation, variability in interpretation, scoring protocol and choice of antibodies with its related lack of reproducibility. To overcome some of these variables, we used tissue TMA with an autostainer and automated scoring system based on bright-field microscopy imaging coupled with advanced colour detection software.

## Conclusion

N-cadherin expression is associated with adverse clinicopathologic features and higher probability of recurrence on univariable analyses in patients undergoing RNU for UTUC. However, when adjusted to the effect of standard prognostic factors on multivariable analyses, this effect disappears, limiting its role in decision-making. Finally, more efforts should be made to find out markers to be incorporated in prognostic schemes in order to help in clinical decision-making process at each UTUC disease state.

## Electronic supplementary material

Below is the link to the electronic supplementary material.

Supplementary material 1 (DOCX 12 kb)
