# Supplementary material for: Prognostic role of expression of N-cadherin in patients with upper tract urothelial carcinoma: a multi-institutional study
Source: World J Urol. 2016 Nov 9;35(7):1073–80. doi: 10.1007/s00345-016-1968-2 (PMC5486535; doi:10.1007/s00345-016-1968-2)
Supplement: Supplementary file 1 — Supplementary material 1 (DOCX 12 kb) [file 345_2016_1968_MOESM1_ESM.docx]

Supplementary table (1)

|  | N-cadherin + | N-cadherin - |
| --- | --- | --- |
| E-cadherin - (325) | 133 (40.9) | 192 (59.1) |
| E-cadherin + (353) | 159 (45) | 194 (55) |

Supplementary table (2)

| **Variable** | **RFS** | | | **OS** | | | **CSS** | | |
| --- | --- | --- | --- | --- | --- | --- | --- | --- | --- |
|  | **HR** | **95%CI** | **p** | **HR** | **95% CI** | **p** | **HR** | **95% CI** | **p** |
| N-cad in(E -) subgroup | 1.60 | 0.98-2.60 | 0.06 | 1.03 | 0.68-1.55 | 0.9 | 1.51 | 0.91-2.54 | 0.1 |
| N-cad in(E+) subgroup | 1.33 | 0.91-1.96 | 0.1 | 0.97 | 0.69-1.36 | 0.9 | 1.23 | 0.81-1.86 | 0.3 |
